# A Stubborn Case of Chlorosis, Accompanied by Menstrual Disorders

**Published:** 1885-07

**Authors:** J. C. Solomon

**Affiliations:** Perry, Georgia


### A STUBBORN CASE OF CHLOROSIS ACCOMPANIED BY MENSTRUAL DISORDERS.

BY J. C. SOLOMON, A. M., M. D., PERRY, GEORGIA, READ BEFORE THE MEDICAL ASSOCIATION OF GEORGIA, AT ITS THIRTY-SIXTH ANNUAL SESSION, AT SAVANNAH, APRIL, 1885.

Miss E. G., age 16, summoned me to her bedside on the 26th of January last. When I arrived I found her almost in an exhausted condition—temperature 100, circulation 120; face full and œdematous, with a dropsical tendency, having a remarkably pale, greenish cast; eyes of a slightly yellow, bilious hue, and of the saddest, most forlorn expression imaginable. There was not the slightest mark of animation in them. So helpless seemed her form; so lifeless her pale, green face; so sad her weary eye, I could not help but stand for a few moments by her bed-side and pity her—pity her with my heart. I listened to her sying respirations and marked her troubled groans. I verily believe I have never seen a sadder picture, a more utterly desolate expression. She was confined to her room for several days, having taken her bed one day previous to my summons. On close examination and inquiry, I found, in addition to the above-mentioned symptoms, much pain in various parts of the body, being especially tender and sensitive to the "touch" along the intercostal spaces, over the sternum and in the region of the clavicles; a dull, heavy throb in the temple, veins being greatly distended here, as well as in the cervical region. On pressure much pain was experienced near the seventh cervical vertebra, along the lumbar column, in the solar plexus of nerves—indeed, all over the body where the nerves come near the surface. On much pressure, not only was considerable pain evinced, but a general sense of uneasiness pervaded the whole being. Both body and mind were literally unstrung and "let down."

My patient's appetite was very morbid, desiring articles unwholesome, unnatural and indigestible; craved chalk, lime, dirt, sticks—craved it constantly; digestion much impaired, bowels constipated, tongue coated, extremities cold and nervous, slight palpitation of the heart, with occasional paroxysms of tremor and great restlessness, considerable aversion to company, preferring silence and solitariness; she could not tolerate her ordinarily most genial friend—no one save her mother.

I soon discovered, too, that her menstrual flows were sadly purturbed, suffering intensely with amenorrhœa and dysmenorrhœa, accompanied still further by leucorrhœa, which was distressing. After making all other examinations and inquiries necessary, I drew from her pale and languid arm a few drops of venous blood, and, placing it under the microscope, found the watery constituents largely augmented together with an unnatural amount of albumen.

With all these terrible symptoms before me, I unhesitatingly gave my diagnosis as a stubborn case of chlorosis, with complications.

*Cause.*—As best I could learn, the active or exciting cause was a disappointment in love, this young lady being in good health, vigorous in body and lively in disposition a month previous to her illness, at which time her faithful affianced died. After this her mind grew sad, her heart was heavy with the burden of her sorrow, her step was slow and painful, her body became languid, her rosy cheeks grew pale, and her beautiful eyes lost their lustrous glow.

At times her grief would become terrific, and she would be verily engulfed in a flood of tears. These sad ravages preyed heavily upon her body and mind, until finally she was overcome with grief and in this pitiable condition I found her.

My first idea was to palliate her diseased mind, to comfort her troubled heart, to sooth her anguishing spirit, hence ordered the room made as cleanly and comfortable as it possibly could be made, had taken away everything that could in any way bear upon her sorrow, or tend to remind her of her hapless dead. In other words, I attempted to divert her mind from the real cause of her trouble, the burden of her grief. Next I directed my attention and my remedies to the neurosis. Her mind was greatly disturbed. I directed my treatment accordingly—gave large doses of hydrate of chloral and bromide of potassium, and ordered this continued every three or four hours; also gave pretty freely of the iodide of potassium, and 11-00 grain hyosciam every two or three hours. As general tonics, I advised carb iron, quinine, strychnia, the sponge bath at night and morning, and sulph. morphia, sufficient to promote rest and relieve pain.

On the following day I found the patient in a strange and uncomfortable state, her head nodding and turning, hands tossing, extremities cold, looking wildly out of the eyes, surface hot, mouth and fauces dry, temperature, circulation, respiration all considerably elevated. She raged and tossed and muttered strange things.

This soon passed off and was followed by a spell of hysterical weeping—sighing, with the most painful respirations—finally went off into quite a marked spell of hysterical weeping. I gave large doses of sach. calomel every two hours till she was purged thoroughly, when her circulation and temperature became normal. I then gave her 10 grs. each of quinine and dovers' powders. In less time than an hour she was asleep and in a profuse perspiration. I watched her attentively, and while her sleep was not as peaceful or satisfactory as I had anticipated, yet she was somewhat refreshed after an hour and twenty minutes nap. However, she had not been awake long when she began to toss uneasily over the bed, threw her hands violently, looked scared, had a slight tremor all over, and was more or less hysterical, her extremities became cold again, and once more her condition was alarming. This time I had her placed into a tub of hot mustard water, giving at the same time a drink of whisky and ¼ gr. morphine; had her bathed for ten minutes, rubbed with a coarse towel till she was in a general glow, and then put to bed. In twenty minutes she was sleeping soundly, more so than she had done in a week. I continued my Iod. Pot. and ordered 10 grs. each of Brom. Pot. and Hydrate-Chloral, to be given every three hours till her sleep became natural. With these, together with the general nervine anodynes, I succeeded in keeping her pretty quiet. In conjunction with this treatment, I

put her on Goodwyn's Comp. Syr. Hypophosphites, knowing full well its great efficacy in the treatment of menstrual disorders, having derived two very satisfactory results from its use in several of my mild chlorotic cases.

This treatment I continued in the main for several days, making only such necessary changes as would be suggested from time to time by the arising symptoms. She improved under it slowly, her hysterical spells growing less frequent and her tremors less and less perceptible. Occasionally these paroxysmal troubles would recur, and at times with great violence. While I knew my patient was better, yet the results were not as eminently satisfactory as I desired, so concluded to try what virtue there might be in electricity. I placed one pole of the battery on the solar plexus of nerves, and the other near the seventh cervical vertebra, and gave her a mild charge for five or six minutes, increasing the power at each repeated application. Once or twice I passed a strong current through the lumbar region, thinking perhaps it might excite the diseased uterus into vigor and its usual activity. From time to time I brushed along over the superficial nerves of the body, and after five days constant application of electricity I discontinued it altogether. After my first application of electricity there was no more tremor, no more hysteria, no more cold feet and hands. The bowels became regular and natural. After keeping her on the hypophosphites for a few days, she was dismissed, fully restored to health.

